# Structure and biocompatibility of poly(vinyl alcohol)-based and agarose-based monolithic composites with embedded divinylbenzene-styrene polymeric particles

**DOI:** 10.1186/2194-0517-2-4

**Published:** 2013-02-21

**Authors:** Lydia G Berezhna, Alexander E Ivanov, André Leistner, Anke Lehmann, Maria Viloria-Cols, Hans Jungvid

**Affiliations:** 1 Protista Biotechnology AB, Bjuv, SE-26722 Sweden; 2 Polymerics GmbH, Berlin, D-12681 Germany

**Keywords:** Cryogels, Poly(vinyl alcohol), Agarose, Divinylbenzene-styrene particles, C5a fragment of complement, Fibrinogen

## Abstract

Macroporous monolithic composites with embedded divinylbenzene-styrene (DVB-ST) polymeric particles were prepared by cryogelation techniques using poly(vinyl alcohol) or agarose solutions. Scanning electron microscopy images showed multiple interconnected pores with an average diameter in the range of 4 to 180 μm and quite homogeneous distribution of DVB-ST particles in the composites. Biocompatibility of the composites was assessed by estimation of the C5a fragment of complement in the blood serum and concentration of fibrinogen in the blood plasma which contacted the composites. A time-dependent generation of C5a fragment indicated weak activation of the complement system. At the same time, the difference in fibrinogen concentration, one of the most important proteins in the coagulation system of the blood, between the pristine blood plasma and the plasma, circulated through the monolithic columns, was insignificant.

**Electronic supplementary material:**

The online version of this article (doi:10.1186/2194-0517-2-4) contains supplementary material, which is available to authorized users.

## Background

Macroporous monolithic materials produced from hydrophilic polymers by cryogelation techniques find more and more applications in biomedical science and technology. The areas for material applications are separation and purification of proteins (Dainiak et al. 2004), bioaffinity screening techniques (Hanora et al. 2005), synthesis of adsorbents for water purification (Le Noir et al. 2007), or development of tissue-engineering scaffolds (Bolgen et al. 2007) including those used for wound healing (Dainiak et al. 2010). Cryogelation is a process of gel formation, which takes place in a semi-frozen state. Under such condition, the gel forms in narrow unfrozen zones with high concentrations of reagents. At the same time, the crystals of frozen aqueous medium act as porogens and form large interconnected pores appearing after defrosting (Kirsebom et al. 2009). The cryogelation techniques allow preparation of elastic mechanically stable monolithic matrices easily permeable to aqueous solutions of proteins and suspensions of cells. The monolithic gels exhibit multiple interconnected pores of 1 to 100 μm in diameter, which can be controlled by changing the conditions of synthesis (Kirsebom et al. 2009; Plieva et al. 2007). Various types of monolithic porous polymer structures, MPPS™, produced by cryogelation of hydrophilic polymers were developed by Protista (http://www.protista.se). An attractive feature of the cryogelation technique is the possibility to embed suspended dispersed materials such as polymeric microparticles into the monoliths, which allows for increased absorption capacity and targeting the adsorbents against selected compounds (Ivanov et al. 2012; Koc et al. 2011; &Özg&ür et al. 2011). Recently, polystyrene microparticles were shown to be effective adsorbents for liver toxins such as bilirubin, bile acid, and aromatic amino acids (Weber et al. 2008). In terms of this concept, the composite cryogels with embedded adsorptive microparticles can be considered as promising materials for extracorporeal blood purification and removal of liver toxins, pro-inflammatory factors, xenobiotics, or even cancer cells from the human blood. Apart from extracorporeal blood purification, the removal of toxic metabolites from human or animal plasma is challenging in view of production of cell growth media. In particular, removal of toxic metabolites related to liver failure or pro-inflammatory cytokine tumor necrosis factor is a need for the studies performed with cultured hepatocytes (Saich et al. 2007; Jones and Czaja 1998). The above production processes and research applications are required for adsorbent devices with low resistance to the flow of plasma combined with effective adsorption of various toxins. The permeable monolithic composites suggest an adequate type of the adsorbent.

However, materials that are supposed to be in contact with human organisms or blood plasma must be biocompatible, in particular, concerning their interaction with clotting and complement systems of the blood (Kirkpatrick et al. 1998). Both agarose and poly(vinyl alcohol) (PVA) were earlier used to synthesize biomaterials (Varoni et al. 2012; Paradossi et al. 2003) Correspondingly, the aim of the present study was to assess the possibility of biomedical applications of the composite cryogels based on PVA or agarose with embedded divinylbenzene-styrene (DVB-ST) polymeric microparticles. To achieve this, the estimation of the C5a component of complement and fibrinogen in the human blood serum or plasma during their contact with the above composite materials has been performed. In view of the different chemical structures of the chosen polymers and, consequently, different methods of cryogelation, the difference in their microstructure and interaction with embedded polymeric microparticles can be expected. Thus, another aim of the study was the characterization of the microstructure of monolithic composites.

## Results and discussion

### Microstructure of the composite and non-composite monoliths

**Figure 1:**
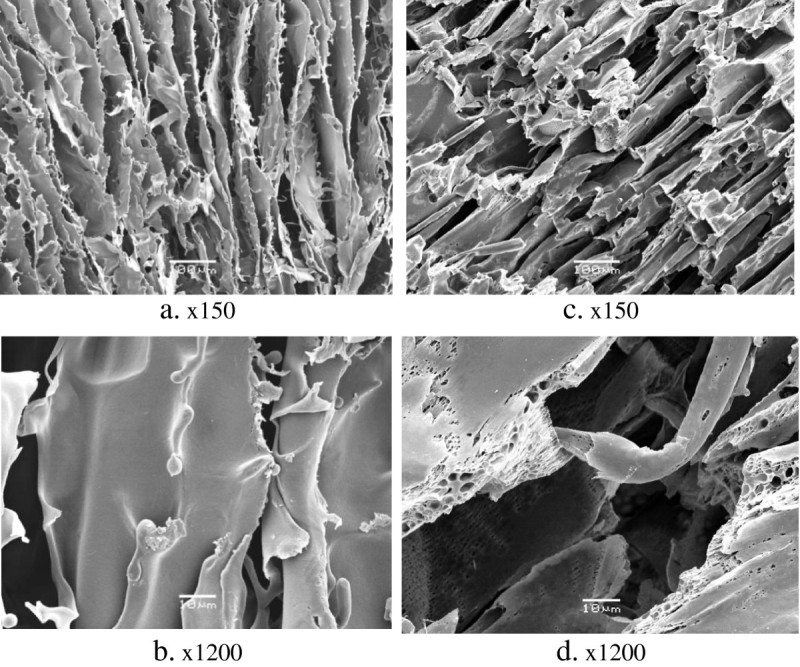
**SEM micrographs of slices of the particle-free monolithic cryogels.** They are prepared from (**a**, **b**) 3% agarose solution and (**c**, **d**) 5% PVA solution.

Micrograph comparison of particle-free agarose and PVA slices, observed by scanning electron microscopy (SEM) at low magnification (Figure 1), has shown that both types of the cryogel monoliths had quite similar structures with evenly distributed interconnected pores, with an average pore size within the range 4 to 100 μm. However, at higher magnification, the difference between the structure of agarose and PVA gels could be observed. The PVA cryogel has porous thick walls of about 10 to 30 μm (Figure 1d) in contrast with the smooth thin (approximately 2 μm) walls of the agarose gel. No visible pores in the walls of the agarose matrix could be seen on the SEM micrographs (Figure 1b). In theory, the different porosities of the particle-free monoliths gels may result in different adsorption characteristics due to an increase in the total binding surface area of the PVA monoliths with porous walls.

**Figure 2:**
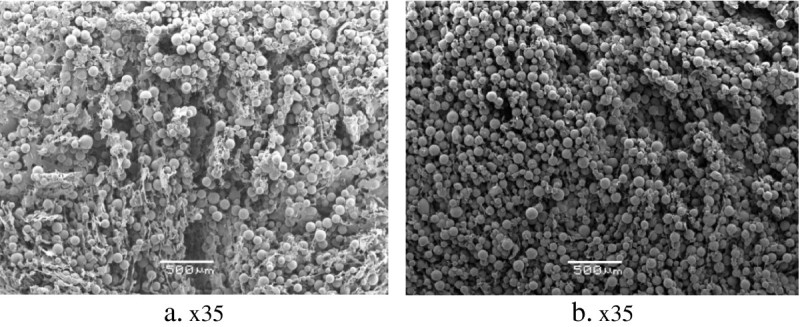
**SEM micrographs of slices of cryogel monoliths.** At (**a**) 3% agarose and (**b**) 5% PVA with embedded DVB-ST polymeric particles.

Both PVA- and agarose-based composites that consist of DVB-ST polymeric particles connected by sheets and bridges of the cryogelated polymers and exhibited large cavities (up to 180 μm in diameter) are detectable throughout the materials, whereas the mean pore size was 30 μm (Figure 2). Some pores of the larger sized particles observed in the composites probably originated from critical point drying, which resulted in 96% shrinkage of the particle-free agarose and PVA, but not of the composites. Resistance of the composites to shrinkage could be explained by the higher mechanical stability provided by the particles.

**Figure 3:**
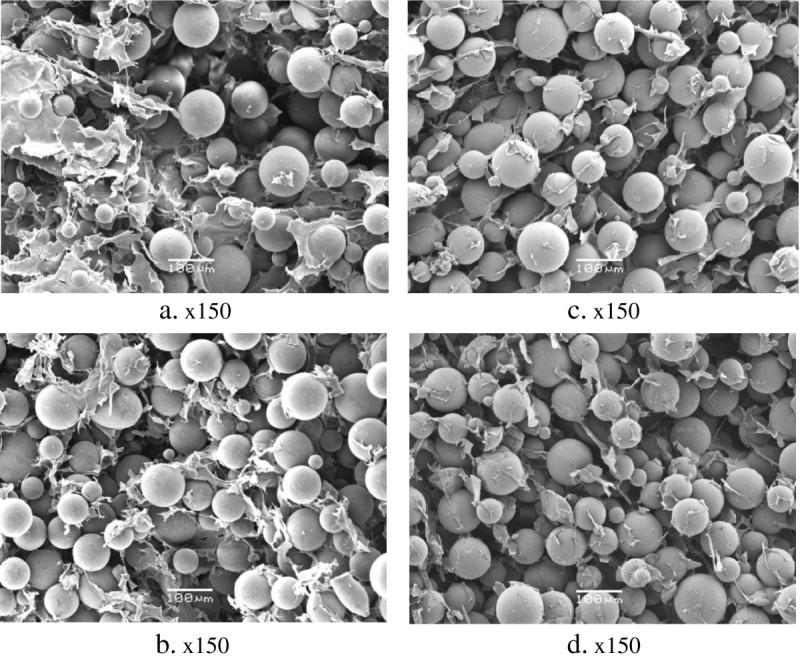
**SEM micrographs of slices of 3% agarose and 5% PVA cryogel monoliths.** (**a**, **b**) 3% agarose and (**c**, **d**) 5% PVA cryogel monoliths with embedded DVB-ST polymeric particles.

Important features of the cryogel composites were high density and homogeneous distribution of the embedded particles in the polymer matrices. There was no big difference between the content of DVB-ST beads in the slices obtained from the top (Figure 3a,c) and the bottom (Figure 3b,d) of the agarose- and PVA-based composites. It is worth noting that some parts of the DVB-ST polymeric particles were tightly embedded or even wrapped in the cryogel matrix, whereas some other parts remained free (Figure 3).

The presence of relatively narrow (4 μm in diameter) pores in cryogel monoliths, as well as the high packing density of DVB-ST polymeric particles, could be a restricting factor for the passage of whole blood through the composites. Some types of leukocytes with sizes of 10 to 21 μm might be stuck in the narrow pores. However, this does not preclude an application possibility of these materials for purification of blood plasma, which could be easily percolated through the monoliths by peristaltic pumping. According to the obtained data, the throughput capacity of the cryogel composites under a pressure of 1 kPa was 0.5 mL/min.

### Adsorption of fibrinogen

**Figure 4:**
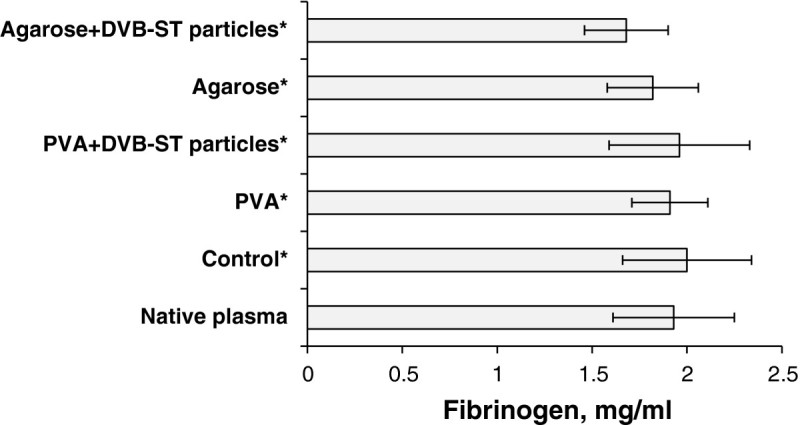
**Fibrinogen concentration in blood plasma (M ± SD;** ***n*** **= 3).** It is percolated through the different types of cryogel monoliths: particle-free 5% PVA, particle-free 3% agarose, PVA, and agarose composites, with embedded (DVB-ST) polymeric particles. The control is plasma percolated through the pumping system without monoliths. Asterisk signifies that *p* ≥ 0.05 in comparison with the native plasma.

For biocompatibility of new biomaterials, determination of fibrinogen as well as some fragments of complement called ‘split products’ in the contacting blood is a mandatory test according to the ISO regulatory documents (ENSAI 2009). Fibrinogen is one of the most important proteins in the coagulation process. Absorption of the fibrinogen homodimer on the surface of any material in contact with blood can mediate the formation of platelet aggregates, further leading to thrombosis (Vogler and Siedlecki 2009; Beugeling 1979). Concentration of the fibrinogen in blood plasma samples before and after their passage through the monoliths, both noncomposite and composite, is illustrated in Figure 4.

Judged by the obtained data, there was no statistically significant depletion of fibrinogen from the blood plasma which contacted the composites in comparison with the plasma passed through the pumping system without monoliths. The low adsorption of fibrinogen on the monoliths may be explained by two independent reasons. The first one is that this protein is known to be rapidly (within 1 to 2 min) adsorbed by a number of artificial materials, which then gradually release the protein in the plasma (Brash et al. 1988). This phenomenon is due to the displacement of fibrinogen by the other plasma proteins, and it is called the Vroman effect (Brash et al. 1988; Vroman et al. 1980). The equilibrium amount of fibrinogen adsorbed from the blood plasma on nonporous polystyrene was found to be as low as 250 ng/cm^2^ (Tsai et al. 1999). This could be a reason why the fibrinogen concentration in the samples of plasma percolated through the monoliths for 30 min was almost the same as that found in the native plasma. The second reason is that the embedded DVB-ST microparticles might have too little fraction of pores accessible for the adsorption of fibrinogen, a relatively large protein with a molecular weight of 340 kDa, and a hydrodynamic radius of 12.7 nm determined at physiological conditions (Wasilewska et al. 2009). The latter hypothesis can be checked by the following calculation. The outer specific surface area of 75-μm DVB-ST beads of known bulk density (0.28 g/cm^3^) can be estimated as *ca*. 0.14 m^2^/g. Note that the total specific surface area of the beads (*S*_BET_ = 641 m^2^/g) is 4,500 times larger due to the inner porosity of the beads (Table 1). Thus, the DVB-ST beads contained in a composite cryogel monolith (500 mg, see the ‘Methods’ section) might have a maximum outer surface area of 0.07 m^2^ available for this protein adsorption. One can evaluate the corresponding equilibrium amount of fibrinogen adsorbed on the outer surface as *ca*. 180 μg/monolithic gel. Under the conditions of the experiment and at ±0.3 mg/mL standard deviation of fibrinogen concentration measurements (see Figure 4), the mentioned adsorbed amount would be within the limits of the experimental error (±1 mg fibrinogen/gel). Since there were no statistically confident differences between the fibrinogen concentrations in the native and the percolated plasma samples (Figure 4), neither the pores of the DVB-ST beads nor the cryogelated polymer structures (PVA or agarose) (see Table 1) could provide essentially more binding sites for fibrinogen than the outer surface of the beads. The composite monoliths adsorbed much lower amounts of fibrinogen than one could expect from DVB-ST polymeric particles with the above specific surface area and a pronounced fraction of macropores (*V*_macro_ = 0.22 cm^3^/g, Table 1). This could be a consequence of low accessibility of pores or, possibly, shielding of the bead surface by microscopic fragments of cryogelated PVA or agarose. Fibrinogen adsorption is known to mediate adhesion of platelets, the phenomenon involved in the formation of thrombus (Pulanic and Rudan 2005). The insignificant fibrinogen adsorption on the composite cryogel monoliths can indicate low thrombogenicity of the materials, though this requires for a more detailed investigation of the blood coagulation system brought into contact with the composites.

**Table 1:** **Characteristics of DVB-ST particles**

Characteristic	Value
Particle diameter (*d*_p_)	75 μm
Specific surface area (*S*_BET_)	641 m^2^/g
Volume of micropores (*V*_micro_)	0.27 cm^3^/g
Volume of mesopores (*V*_meso_)	0.88 cm^3^/g
Volume of macropores (*V*_macro_)	0.22 cm^3^/g
Average pore diameter (*d*_pore_)	7.7 nm

### Activation of complement system

**Figure 5:**
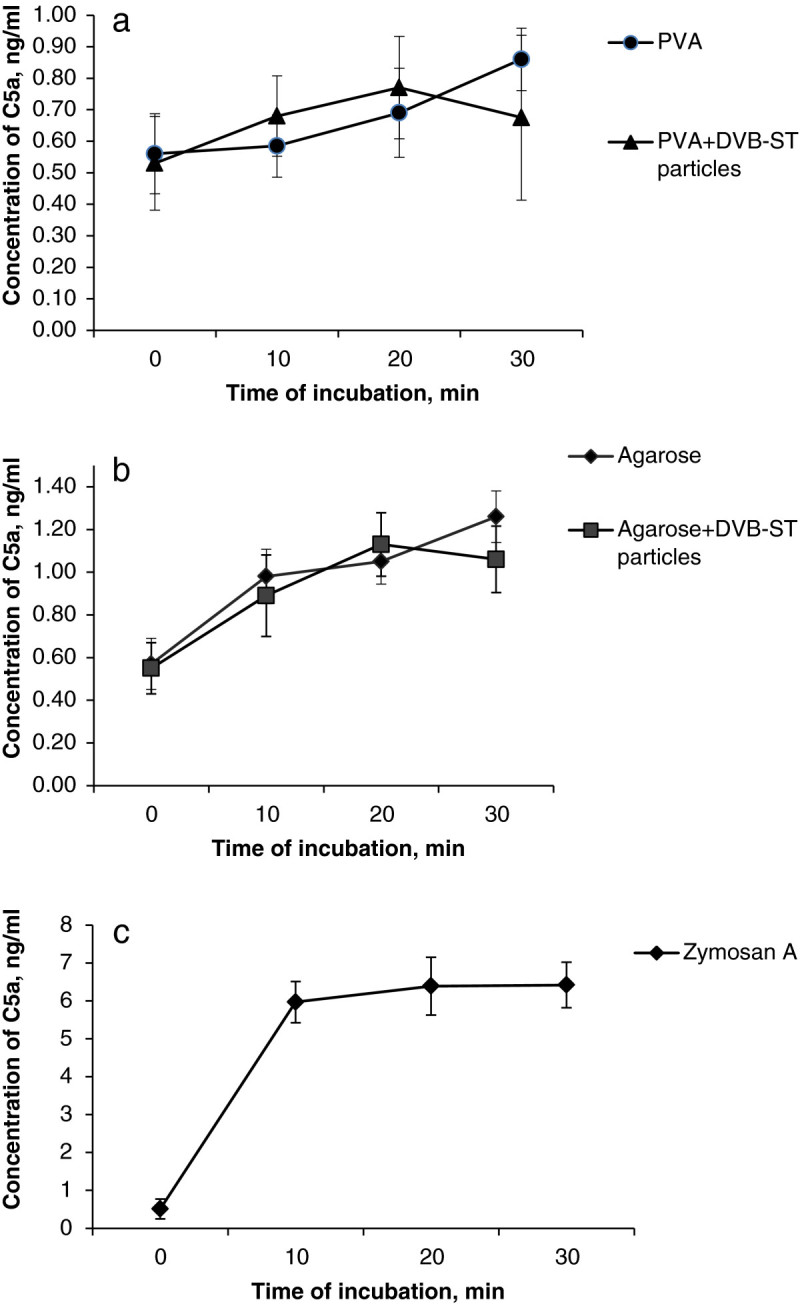
**Generation of C5a fragment in the human serum (M ± SD;** ***n*** **= 3).** This took place after incubation with four different types of cryogels: (**a**) slices of particle-free 5% PVA cryogel and PVA composite embedded with DVB-ST polymeric particles, (**b**) slices of particle-free 3% agarose and agarose composite with embedded the DVB-ST polymeric particles, and (**c**) positive control (zymosan A, 4 mg/mL in serum). Asterisk signifies that *p* ≤ 0.05 in comparison with the positive control.

Determination of C5a is one of the methods for the estimation of complement system activation that resulted from the contact of blood with artificial materials (ENSAI^2009^). C5a anaphylatoxin, formed as a part of the sequential cascade of complement activation pathways, is known as a potent pro-inflammatory agent, which takes part in leucocyte activation, cytokine production, release of histamine etc. (Sarma and Ward 2010). In the present study, the time-dependent generation of C5a in the human blood serum during its contact with particle-free or composite monoliths has been examined. The concentration of C5a in the serum incubated with slices of particle-free PVA and agarose cryogels increased with time and, at the final point (30 min), was 1.5 to 2 times higher than the initial value (Figure 5a,b). These data demonstrated that both macroporous gels of agarose and PVA activated the complement system in the human serum, which is in agreement with earlier reported data (Lyle et al. 2010; Black and Sefton 2000; Arima et al. 2009) where the complement activation through the alternative pathway by such polymers as agarose (Lyle et al. 2010) and PVA (Black and Sefton 2000) hydrogels has been shown. In those experiments, the concentrations of the Bb fragment and the soluble form of the terminal membrane attack complex SC5b-9 were measured during the contact of hydrogels with blood serum (Black and Sefton 2000). It is relevant to note that hydrophobic polymers such as polyethylene or polydimethylsiloxane brought into contact with serum caused a much weaker activation of the complement compared with that in hydrophilic polymers such as PVA or cellulose. Heavily hydroxylated chains of these polymers are believed to trigger the alternative pathway. In our experiments, complement activation due to contact of serum with the composite monoliths seemed to be relatively weak because the positive control serum containing zymosan A exhibited a much higher 11-fold increase in the concentration of C5a, already after 10 min of incubation (Figure 5c).

There was no statistically valid difference between the time dependent generation of C5a fragment in the serum contacting the particle-free cryogels and the composites (Figure 5a,b). A slight decrease in C5a concentration, observed for both composites after 30 min of the contact, can be explained by adsorption of the C5a fragment on the embedded DVB-ST polymeric particles. It seems like the observed activation of the complement was mainly due to the interaction of its components with the hydrophilic PVA and agarose.

## Conclusions

Macroporous monolithic composites based on cryogelated PVA and agarose with embedded DVB-ST microparticles were prepared. The composites were densely loaded with homogeneously distributed microparticles and exhibited multiple interconnected pores with the size of 4 to 180 μm. The porous structure allowed for a facile percolation of blood plasma through the monoliths. The percolation did not influence fibrinogen concentration in the plasma, which indicated the absence of any significant effect of the materials on the blood coagulation system. The contact of both particle-free and composite monoliths with blood serum caused a slight increase in the C5a concentration in the serum, which was mainly due to the interaction of complement components with cryogelated PVA and agarose. Embedment of DVB-ST polymeric microparticles into the cryogel monoliths may be advised for the further investigation of these composites as a promising substance for the synthesis of blood compatible materials.

## Methods

### Materials

Poly(vinyl alcohol) with an average molecular weight of 1.3 × 10^5^ g/mol (Mowiol 18–88) was purchased from Clariant GmbH (Frankfurt-on-Main, Germany). Agarose (electrophoretic grade) was from Fluka (Buchs, Belgium). DVB-ST polymeric particles (*d*_p_ = 75 μm) were produced by Polymerics GmbH (Berlin, Germany); their characteristics are listed in Table 1. Glutaraldehyde (GA) (50% aqueous solution), zymosan A from *Saccharomyces cerevisiae*, and other chemicals were obtained from Sigma-Aldrich (St. Louis, MO, USA) or Merck (Darmstadt, Germany). The commercially available ELISA kits from GENTAUR (Kampenhout, Belgium) and CUSABIO (Wuhan, China) were used for immunoassays of fibrinogen and C5a fragment of complement in the blood plasma and serum, respectively.

### Methods

#### Preparation of cryogel monoliths

Cryogel-based monoliths were prepared in plastic syringes according to the earlier reported technique (Plieva et al. 2006). In brief, PVA-based monoliths (3.2 cm in length and 0.9 cm in diameter) were prepared from pre-cooled 5% PVA aqueous solution adjusted to pH 1.0 to 1.2 with 1 M HCl by cryogelation in an air bath cryostat (Arctest, Finland) at −12°C overnight. GA at a concentration of 1% was used as a cross-linker. Decimolar aqueous solution of NaBH_4_ was circulated through the columns by peristaltic pumping for the blocking of residual aldehyde groups. The monoliths treated in that way were washed with distilled water to neutralize pH, placed into 30% *v*/*v* aqueous ethanol solution, and stored in a fridge at +4°C.

Agarose-based cryogel monoliths were prepared from 3% agarose solution, adjusted to pH 12 with 5 M NaOH. The alkaline agarose solution pre-warmed in a water bath at 60°C was placed into a liquid bath cryostat (Lauda, Germany) at −32°C for 30 min for fast freezing and then kept in the air bath cryostat at −12°C overnight. After defrosting, all monoliths were washed with distilled water to neutralize pH, placed into 30% ethanol solution, and stored in a fridge at 4°C. Before the contact with blood serum or blood plasma, the cryogel monoliths were washed with distilled water and PBS (pH 7.4).

In order to synthesize the composite monoliths, DVB-ST polymeric particles were washed with 2-propanol, distilled water, suspended in 5% *w*/*v* PVA or 3% *w*/*v* agarose solutions, and rinsed with the same solutions. The suspensions were adjusted to 250 mg/mL concentration of DVB-ST particles in the polymer solutions, which were put through cryogelation as described above.

#### Scanning electron microscopy examination

The monoliths have been examined under a scanning electron microscope (JEOL JSM-5600LV, JEOL Ltd., Akishima, Tokyo, Japan). In order to prepare the samples for SEM, the top and bottom of each cryogel monolith were cut off to get 4-mm-thick slices, which were further incubated in 2.5% GA solution in 0.12 M sodium phosphate buffer (pH 7.2) overnight. The samples were dehydrated by soaking in aqueous ethanol of increasing concentration (from 0% to 99.5% *v*/*v*), critical point dried, and coated with gold/palladium (40:60).

#### Preparation of blood plasma and serum

Blood samples were taken from two voluntary healthy donors into special plastic tubes to obtain the blood serum or plasma (1 mL of 0.1 M sodium citrate to 9 mL of blood). Tubes with the blood without anticoagulant were immediately placed in a water bath at 37°C for clot formation. Afterwards, all test tubes were centrifuged for 10 min at 5,000 rpm; serum and/or plasma were aspirated, pooled into the plastic Falcon tubes, and placed in a fridge at 4°C.

#### Percolation of blood plasma through the monoliths and fibrinogen assay

Fibrinogen concentration in blood plasma was measured before and after its circulation through the cryogel monoliths. Plasma (3.6 mL) was circulated through each of the tested cryogel monoliths (0.9 cm × 3.2 cm) by peristaltic pumping (WATSON MARLOW 101 U, Sweden) at 0.3 mL/min flow rate for 30 min. Instruction from the commercially available column for extracorporeal blood purification TORAYMYXIN PMX-20R (Toray Industries, Tokyo, Japan) has been used for calculations of monolith parameters, time of circulation and volume of plasma, passing through (Toray Industries 2007). Control plasma was passed through the empty syringe. Having been passed through monoliths, the plasma samples were stored at −20°C and assayed after defrosting according to the manual for the ELISA kit.

#### Contact of blood serum with monoliths and C5a assay

Monoliths sliced into 3-mm-thick disks were incubated in the blood serum (1 mL) for 0, 10, 20, and 30 min (37°C, water bath). Having been incubated with monoliths, serum was frozen, stored at −20°C, and assayed after defrosting according to the manual of the ELISA kit. Zymosan A prepared as described earlier (Craddock et al. 1977) was used at a concentration of 4 mg/mL in the serum as a positive control for the complement system activation. According to the data presented in the literature, the concentration of zymosan required for complement activation *in vitro* is in the range of 1.0 to 5.0 mg/mL (Craddock et al. 1977; Yuanyuan et al. 2001; Fehra and Jacob 1977).

### Statistical analysis

All calculations and construction of calibration curves were performed using the Origin (Microcal Software Inc., Northampton, MA, USA) and Excel (Microsoft Corporation, Redmond, WA, USA) programs. Significant difference between each two different groups of data was examined using the Student's *t* test.

## Authors’ original submitted files for images

Below are the links to the authors’ original submitted files for images. Authors’ original file for figure 1 Authors’ original file for figure 2 Authors’ original file for figure 3 Authors’ original file for figure 4 Authors’ original file for figure 5 Authors’ original file for figure 6
