# Supplementary material for: Structure and biocompatibility of poly(vinyl alcohol)-based and agarose-based monolithic composites with embedded divinylbenzene-styrene polymeric particles
Source: Prog Biomater. 2013 Feb 21;2:4. doi: 10.1186/2194-0517-2-4 (PMC5151119; doi:10.1186/2194-0517-2-4)
Supplement: Supplementary file 6 — Authors’ original file for figure 6 [file 40204_2012_7_MOESM6_ESM.docx]

Figure 6. Generation of C5a fragment in the human serum (M ± SD; n = 3), after incubation with the four different types of cryogels: (a) slices of particle-free 5 % PVA cryogel and PVA composite embedded with DVB-ST particles; (b) slices of particle-free 3 % agarose and agarose composite with embedded the DVB-ST particles; (c) positive control (Zymosan A, 4 mg/ml in serum).

* - p ≤ 0.05 in comparison with the positive control.
